# A proposal on bird focal species selection for higher tier risk assessments of plant protection products in the EU

**DOI:** 10.1093/inteam/vjae048

**Published:** 2025-01-06

**Authors:** Benedikt Gießing, Steven Kragten, Ines Hotopp, Anja Russ, Marie Fan, Dennis Sprenger, Arnd Weyers, Christian Wolf

**Affiliations:** tier3 solutions GmbH, Leverkusen, Germany; Syngenta Agro GmbH, Frankfurt, Germany; tier3 solutions GmbH, Leverkusen, Germany; tier3 solutions GmbH, Leverkusen, Germany; BASF SE, Limburgerhof, Germany; Corteva Agriscience, München, Germany; Bayer CropScience, Monheim am Rhein, Germany; tier3 solutions GmbH, Leverkusen, Germany

**Keywords:** EFSA guidance, probability of exposure, higher tier risk assessment, focal species selection, farmland birds

## Abstract

The revised EFSA 2023 Guidance on the risk assessment of plant protection products for birds and mammals emphasises vulnerability as a relevant criterion for focal species (FS) selection rather than prevalence. The EFSA 2023 Guidance suggests to rank FS candidates for each dietary group according to their expected exposure by estimating a species-specific daily dietary dose (DDD). Species experiencing higher exposure would be ranked as potentially more vulnerable and can be identified as FS candidates. The DDD is calculated using an estimated “proportion of diet an individual obtains from the (treated) crop” (PT). A PT is derived from a radio-tracking field study in the crop of interest, but such data are not available for all species. We introduce the frequency of occurrence in surveys (FO_survey_) in each study field from FS field studies as a proxy for PT in theoretical DDD calculations. The presence of a species during a high proportion of surveys, resulting in a high FO_survey_, could indicate a high proportion of foraging time spent in this crop. To evaluate whether FO_survey_ is an appropriate proxy for PT, empirical PT values from radio-tracking studies for different bird species were correlated to respective FO_survey_ values from FS studies in the same crop and growth stage. Based on 10 case examples covering different species and crops, a positive correlation was shown between PT and FO_survey_, supporting the suitability of the proposed approach. Based on a positive correlation between the species’ prevalence and the new theoretical DDD, the list of the most relevant FS resulting from the new ranking approach is not expected to differ significantly from the FS selection, according to the methodology proposed in EFSA 2009. However, in a few cases, additional species were identified as potential FS, therefore requiring further consideration in the risk assessment.

## Teaser

**Key points**

- The revised EFSA 2023 Guidance Document on the risk assessment of plant protection products for birds and mammals emphasizes vulnerability as a relevant criterion for focal species selection rather than prevalence, but provides unsatisfactory guidance on how this should be implemented.
- Here, we investigate an approach that uses the frequency of occurrence in surveys from focal species studies for daily dietary dose calculations to rank species according to their vulnerability to exposure and select the most vulnerable species as focal species.
- Based on 10 case examples covering different species and crops, the suitability of the proposed approach was shown.
- The most relevant focal species resulting from the new ranking approach are not significantly different from the focal species selection according to the methodology in EFSA 2009; only in a few cases, additional species were identified as potential focal species and require further considerattion.

## Introduction

For the registration of plant protection products (PPPs) in the European Union (EU), it is required that registrants demonstrate an acceptable risk to nontarget organisms, including birds, following the proposed use of the PPP in a certain crop. For birds, the European Food Safety Authority (EFSA) has produced guidance documents (GDs) on how such a risk assessment should be conducted (EFSA, 2009; EFSA et al., 2023). The risk assessment follows a tiered approach. In the lower tier, conservative default assumptions are used, which should filter out low risk PPPs from further risk assessment. In case a lower tier risk assessment fails to demonstrate an acceptable risk, a higher tier risk assessment can be conducted, in which more realism is introduced.

In EFSA et al. (2023), which updates the 2009 birds and mammals risk assessment guidance document, lower tier risk assessments are based on so-called generic model species (GMS). These are not real species, but they represent a potential species occurring in the crop at the time of application of the PPP. For example, a GMS can be a “small omnivorous bird” with an arbitrarily selected but indicative body weight of 27 g and a representative diet containing 50% arthropods, 25% seeds, and 25% monocotyledonous foliage. These proportions of the diet composition are given for various crop scenarios and are more variable in EFSA et al. (2023) than the previous guidance (EFSA, 2009). When the risk assessment using this GMS fails, a higher tier risk assessment can be conducted using real focal species (FS). These are actual species occurring in the crop at the time of PPP application. Real FS are selected to be representative of worst-case exposure scenarios, ensuring that the FS are vulnerable to PPP exposure relative to the product use pattern considering the relevant crop and growth stage due to their dietary habits, foraging behavior, and habitat use. The real FS are intended to be protective for other species within the same dietary guild (e.g., granivorous, herbivorous, insectivorous, omnivorous). This ensures that the species chosen for the risk assessment is indicative of the broader group and should be protective for all other species within this respective guild.

The vulnerability of a species takes the probability of exposure, toxicological sensitivity, and potential for recovery into account (EFSA Scientific Committee, 2016). Here, we focus on the probability of exposure as one aspect of vulnerability. To be exposed to a PPP, a real FS should be to some extent prevalent and abundant in the field of crop of interest.

Following EFSA (2009), real FS should be determined via dedicated field studies using specific criteria. According to EFSA (2009), species with a frequency of occurrence (i.e., percentage of investigated fields on which the species was recorded, hereafter FO_field_) above 20% might be considered to be of relevance especially if they have high dominance (i.e., make up a relative large part of the total avian community observed). Here, FO_field_ refers to the proportion of study fields where the species was observed, indicating its prevalence across the study area. In contrast, FO_survey_ refers to the proportion of surveys per field where a species was observed, which reflects the extent to which the species utilizes the specific study fields. Furthermore, factors like feeding strata, food intake rate, body weight, and diet should also be considered to ensure that species with the highest potential exposure to the PPP are included in the final risk assessment. Over the years, PPP producers have conducted many such studies to determine FS for a wide range of crops in the EU (Bonneris et al., 2018; Dietzen et al., 2014).

The updated birds and mammals guidance document (EFSA et al., 2023) revised the process of selection of real FS. The key difference between the selection criteria in EFSA 2009 and EFSA et al. 2023 is that FO_field_ > 20% is no longer a relevant criterion, and a low FO_field_ should not eliminate a species from consideration out-of-hand. According to EFSA et al. 2023, a vulnerable species that is infrequently observed but nevertheless clearly present may be considered a more appropriate FS for the risk assessment, and more protective of similar species, than a less vulnerable species with high occurrence. Consideration of infrequently observed species, thus with a low FO_field_ (i.e., species observed in just a few fields of the crop) means that it will be difficult to define an FS, as potentially every species recorded in the crop may be considered of relevance for the higher tier risk assessment. However, exposure is influenced not only by the frequency of occurrence but also by the duration of presence in the crop. Therefore, selecting an infrequently occurring species with a low body weight (and correspondingly high food intake rate), might not result in a risk assessment that covers the risk for other species during the time of a PPP application as well. For example, the Eurasian wren (*Troglodytes troglodytes)* is a small passerine with a body weight of about 8.3 g (Dunning, 2008) and might occasionally be observed in cropped fields. However, the species predominantly uses a variety of (semi-)natural habitats and is unlikely to spend significant foraging time in recently treated fields (Cramp, 1988). Consequently, it is not expected that this species will be more exposed to PPPs than other species, despite its very low body weight, as its active time spent in recently treated fields and thus the proportion of diet obtained in the crop under assessment (PT) can be assumed to be low. This example underlines the importance of clarifying what is meant by “infrequently observed but nevertheless clearly present,” as this term is not quantitatively defined in EFSA et al. (2023). “Clearly present” likely refers to species observed more than once during a study or application period, excluding chance findings, and should take the ecology of the species into account. Additionally, FS and PT studies must be conducted at the relevant BBCH (Biologische Bundesanstalt, Bundessortenamt und Chemische Industrie) growth stage for the specific PPP application to ensure their accuracy and relevance. Although EFSA appears to move away from absolute thresholds, such as FO_field_ > 20%, a well-designed study with sufficient fields and observations conducted at the appropriate BBCH stage should provide clarity on whether a species can be considered clearly present without requiring a strict quantitative definition. Nevertheless, the ecological context and species' behavior remain critical for interpreting these observations. A precise definition of clearly present goes beyond the scope of this work and would need to be established primarily by EFSA to ensure consistency across risk assessments.

To contribute to a harmonized approach for focal species selection, we propose a method based on the updated EFSA et al. 2023 guidance, which emphasizes the importance of covering potentially vulnerable species based on theoretical exposure. As outlined in EFSA et al. 2023, FS candidates are ranked according to their expected magnitude of exposure by calculating a species-specific daily dietary dose (DDD). With this, species experiencing a relatively higher exposure are ranked as potentially more vulnerable and are identified as FS candidates. This DDD is calculated using body weight, food intake rate (FIR), and the “proportion of diet an individual (potentially) obtains from the treated crop” (PT). A PT is generally derived from a radio-tracking field study, which measures the active foraging time of a species in the field (e.g., Ludwigs et al., 2017). However, these data are not available for all species and crops. Therefore, we propose to use FO_survey_, the proportion of surveys in which a species was observed in each study field obtained from FS field studies, as a proxy for PT in theoretical DDD calculations.

The FO_field_ illustrates the species’ prevalence and distribution rather than its utilization of the crop. Hence, those species that are abundant and widespread are in general more likely to reach a high FO_field_ than rarer and/or more patchily distributed species. In contrast, the FO_survey_ (proportion of surveys per study field where a species was observed) gives a much better indication of the extent a certain field was used by a species. Hence, even rarer and/or more patchily distributed species occurring in just a few study fields (i.e., only reaching a low FO_field_) can be identified as using these fields intensively. The presence of one or several individuals of a species during a high proportion of surveys, resulting in a high FO_survey_, could therefore be an indication of high utilization and, thus, a potential high proportion of foraging time in this crop.

To evaluate whether FO_survey_ is indeed an appropriate proxy for PT, empirical PT values acquired from radio-tracking studies for different bird species were correlated to the respective FO_survey_ values for these species obtained in previously conducted FS studies in the same crop and BBCH growth stage. For this, PT and FS field studies conducted in different crops and BBCH stages for regulatory purposes were used as the data source. Then, species were ranked based on FO_survey_ and the outcome was compared with ranking species based on FO_field_ according to EFSA (2009).

## Methods

### Field study data

Data used for this project were obtained from several industry-owned field studies on bird focal species (FS studies) and PT (PT studies) conducted in the EU during the years 2006 to 2021. Out of a total of 44 FS and PT studies initially considered, we identified 12 suitable FS-PT study pairs. To form a pair, the FS and PT studies had to be conducted in the same region of a regulatory zone, in the same crop, and at the same crop growth stage (BBCH stage). These 12 FS and PT study pairs were aggregated into 10 individual datasets because in two cases, two PT studies were paired with the same FS study. This was done to include one additional relevant data point for the PT. An overview of the regulatory zone, crop, and BBCH stage of these studies is given in the supplemental material (See online supplementary materialSupplement 1; Table 1). For the analysis, the data were anonymized for reasons of confidentiality by replacing crop and species names with generic names. Only bird species assigned to the insectivorous, granivorous, and herbivorous feeding guilds were taken into account. No mixed diet was considered. Omnivorous species were assigned to the respective feeding guilds assuming that they forage entirely on items of each of these guilds. This simplification is necessary because reliable information on the dietary composition of omnivorous species is not always available. Therefore, single diets represent worst-case assumptions and are used for the omnivorous species, although mixed diets are also considered in the EFSA guidance (EFSA et al., 2023).

**Table 1.** Differences between datasets obtained from focal species (FS) studies using the transect count or scan sampling method.

	Transect count	Scan sampling
**Number of surveys per field**	3–4	> 75 (often > 1,000)
**Time between surveys**	At different BBCH growth stages (2 to 8 weeks between surveys)	At the same or similar BBCH growth stages (4–20 days between surveys)
**Mean number of fields**	22	13
**Method**	Actively going through the field	Observation from one or multiple points
	Disturbance of the birds	No disturbance of the birds
	Hidden birds that are flushed are recorded	Very small distantly located birds might be overlooked
	Possible at all crop stages	At the field margin. Not suitable for higher crop stages as birds will not be visible due to developed crop
**Number of studies**	7^a^	3

*Note.* BBCH = Biologische Bundesanstalt, Bundessortenamt und Chemische Industrie.

a Two of the FS studies with transect counts were used in two of the case examples each.

Species in these three feeding guilds were then assigned to four feeding groups according to the food type specified in the guidance: ground dwelling invertebrates, flying and foliage dwelling insects, grains and seeds, and herbs and leaves (See online supplementary materialSupplement 1; Table 2).

**Table 2.** Case I: Results from a focal species study.

	FO_field_ %	FO_survey_ (90^th^ percentile) %	DDD_survey_	PT (90^th^ percentile consumer)
**Species 1**	100.00	97.04	8.19	0.35 (*N* = 20)
**Species 2**	100.00	81.19	7.82	0.39 (*N* = 20)
**Species 3**	75.00	61.30	6.54	0.19 (*N* = 20)
**Species 4**	25.00	49.97	6.32	–
**Species 5**	37.50	28.58	2.71	–
**Species 6**	12.50	2.70	0.30	–

*Note.* All species selected in the newly proposed approach were likely covered by the previously selected focal species. The species ranked highest by the former percentage of investigated fields on which the species was recorded (FO_field_) >20% criterion also ranked highest in the theoretical daily dietary dose (DDD_survey_) approach and thus likely, all relevant species were already selected as focal species. The species are ranked according to their frequency of occurrence in surveys (FO_survey_). Only the six highest ranked species are given here. The full table is available in the online supplementary material. PT = proportion of diet an individual obtains from the (treated) crop.

Bird surveys in the 10 selected FS studies were conducted by using either “transect counts” (Bibby et al., 2000) or “scan samplings” (EFSA et al., 2023). All FS studies were conducted in the southern or central regulatory zone, in leafy vegetables, stone fruits, citrus, maize, oilseed rape, and vineyards spanning all BBCH stages and observing 16 to 41 different bird species using either transect counts or scan sampling as observation method. In transect counts, a predefined transect in a field is walked by one or more observers and birds are detected visually or acoustically. In contrast, scan sampling involves systematically observing a defined part of a field from a fixed point to record all birds present. In the statistical analysis, these two methods were considered separately, because the two approaches differ regarding the mean number of fields surveyed and number of surveys per field (Table 1). Their applicability also depends on the conditions of the habitats. Although the scan sampling approach is most suitable when the visibility of birds in the crops is not prevented by vegetation, it is in principle also possible to conduct transect counts in crops with high vegetation. Whereas open fields with no or low vegetation are preferred by most species only for foraging and not for breeding, higher crops are often used for both foraging and breeding (Cramp & Perrins, 1994). Because the number of surveys per field can be highly variable between studies (Table 2), only studies with at least three surveys per field were included in the analysis. With less than three surveys, only two values of FO_survey_ would be possible for a single field—50% and 100%—and this was considered to be insufficient for a statistical analysis.

The selected 12 PT studies were carried out between 2006 and 2021, following recommendations outlined in EFSA (2009), as the studies were conducted prior to the publication of the revised EFSA Guidance (2023). All PT studies were conducted in the same regulatory zones and in the same crops at the same or similar stages as their FS study counterpart. Between nine and 22 individuals per species were tracked. Up to five avian species were radio-tracked per study to assess the relative use of the crop of interest as foraging habitat by the respective species. All birds were tracked continuously for the full daily activity period. The PT calculations were done in the studies as described in EFSA (2009). Specifically, the 90^th^ percentile for consumers was calculated following the regulatory accepted approach. In case of multiple tracking sessions with the same individual, the mean of the sessions was calculated.

### Data analysis

#### Calculation of FO_field_, FO_survey_, and DDD_survey_

Based on suitable FS-PT study pairs, we calculated the percentage of fields in which a species was observed in a study (FO_field_), the 90^th^ percentile of the percentage of surveys (transect count or scan sampling) in which a species was observed in a study (FO_survey_), and the theoretical DDD (using the FO_survey_ as PT surrogate, DDD_survey_; see below) for each species in the selected FS studies. The 90^th^ percentile of FO_survey_ was chosen to allow for direct comparability with the regulatory accepted 90^th^ percentile of the PT (EFSA et al., 2023). If a species occurs only on a few fields, the FO_survey_ value of this species would be considerably reduced if fields where the species was not observed were included in the calculation even if the species is observed in every survey in the fields where it is present. This could result in its exclusion from further consideration as FS according to the EFSA (2009) guidance, which uses FO_field_ as a criterion. However, species with restricted occurrence especially can still show a high PT value. To account for this, study fields where the species was never observed were excluded from the calculation of the 90^th^ percentile of FO_survey_ for the respective species. This ensures that zero values from irrelevant fields do not artificially lower the 90^th^ percentile of FO_survey_, accurately reflecting the frequent use of specific fields by species with a restricted occurrence. The FO_survey_ was then used in the calculation of the DDD_survey_, which is a function of PT, body weight (bw), food intake rate (FIR), and residues per unit dose (RUD) of the relevant food item (EFSA et al., 2023): 


$${\text{DDD}}_{\text{survey}}={\;\text{FO}}_{\text{survey}}/100\cdot \text{FIR}/\text{bw}\cdot \text{RUD}.$$

The FIR is a function of energy content, moisture content, and assimilation efficiency for specific food items (EFSA et al., 2023). Based on literature and expert knowledge, each species was assigned to a specific feeding guild. Based on its assigned diet and body weight, the FIR/bw value was calculated. Furthermore, diet item–specific RUD values (EFSA et al., 2023) were used to assess the overall exposure. During this process, a species could be assigned to more than one feeding guild. This could be the case if a species has a generally omnivorous diet but shows certain preferences depending on the food availability and nutritional requirements throughout the year, such as the skylark (Holland et al., 2006). Values for RUD, the food energy of a food item, energy content, moisture content, and assimilation efficiency were taken from EFSA et al. (2023). Body weights were taken from Dunning (2008) using either mean body weight or, in case multiple mean values (e.g., separate mean for each sex) were given, the lowest of these mean values was used.

#### Correlation of FO_survey_ (90^th^ percentile) with empirical PT data

A linear regression was calculated to investigate whether FO_survey_ values of species in FS studies correlate significantly with empirically obtained PT values from radio-tracking studies. The data of all examples were pooled. Multiple generalized linear mixed models were tested and the best one chosen based on suitable model diagnostics and the Akaike information criterion (AIC).

#### Correlation of FO_field_ with DDD_survey_

A second linear regression between DDD_survey_ and FO_field_ was calculated for each feeding group to examine whether the newly suggested approach in EFSA et al. (2023) may lead to similar or distinctly different FS candidates compared with the EFSA (2009) selection criteria. Again, the data from all examples were pooled and multiple generalized linear mixed models were tested for each feeding group and the best one chosen based on suitable model diagnostics and the AIC.

All calculations were conducted in R 4.2.2 (R Core Team, 2023) using the“glmmTMB” package (version 1.1.7, Brooks et al., 2017). Further information on the statistical analysis is given in the supporting information (See online supplementary materialSupplement 2).

## Results

### Correlation of FO_survey_ (90^th^ percentile) with empirical PT data

**Figure 1.**
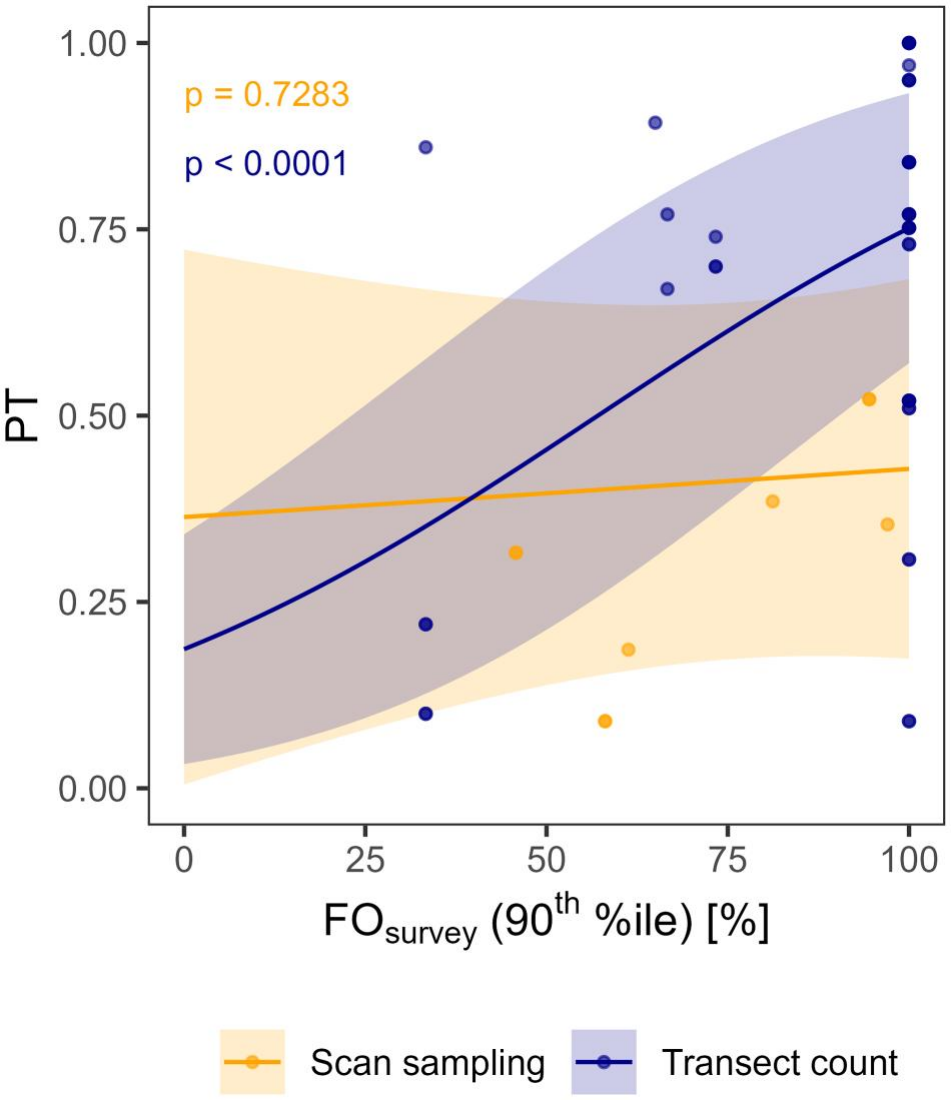
Prediction plot for PT (proportion of diet an individual obtains from the [treated] crop) in relation to the frequency of occurrence in surveys (FO_survey_). Points indicate the original data from scan sampling and transect count studies. Lines indicate the model predictions from the respective generalized linear mixed models and shaded areas show the confidence intervals of the predictions.

Figure 1 shows the relationship between FO_survey_ and empirical PT data separately for the transect count and the scan sampling approach. The results of the best statistical model showed a significant positive correlation between PT and FO_survey_ for the transect count method (*p* < .05; *N* = 35), indicating the potential suitability of the approach to use FO_survey_ as a proxy for PT. For the scan sampling method, this correlation was not significant (*p* = .73; *N* = 9).

### Correlation of FO_field_ with DDD_survey_

**Figure 2.**
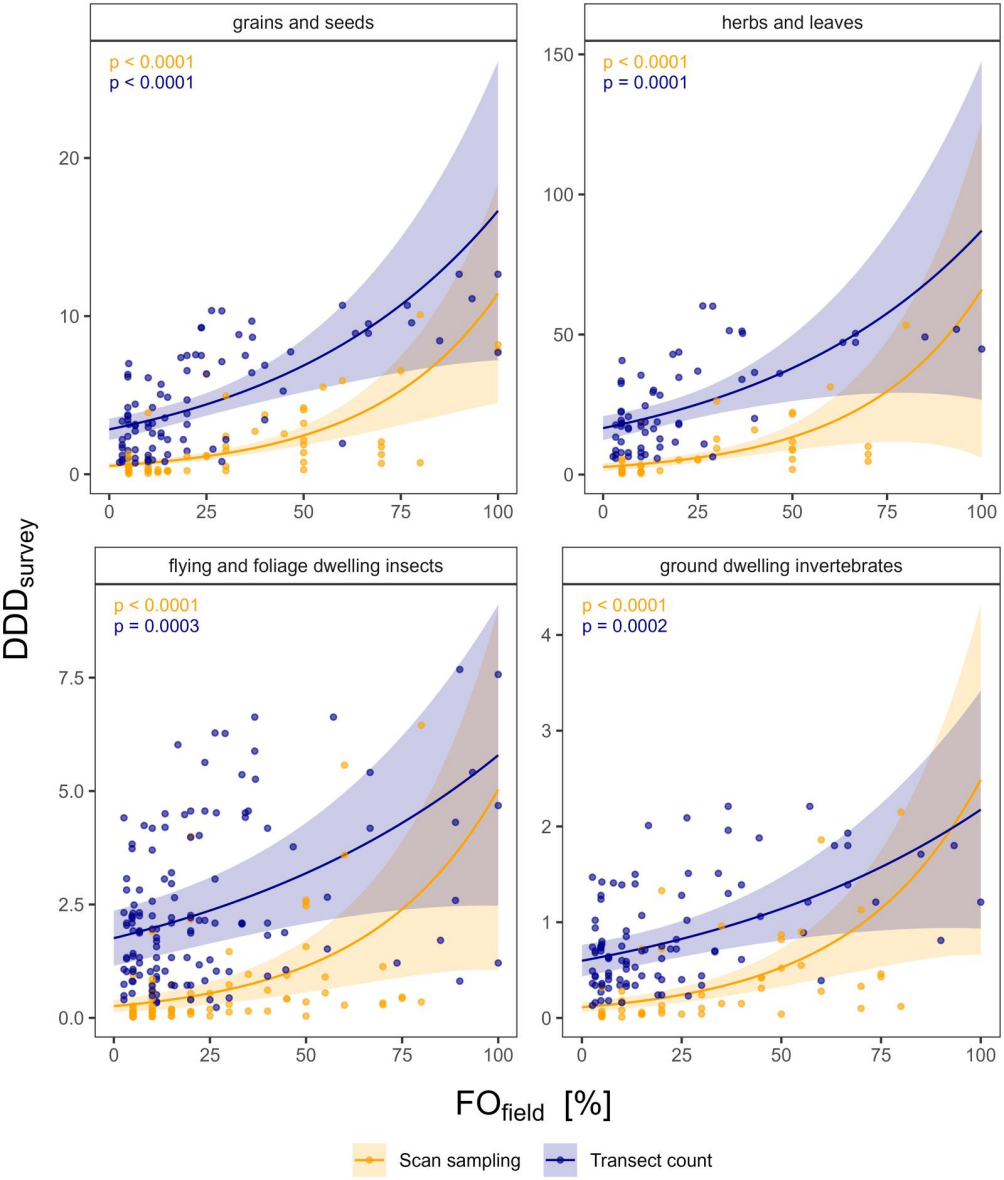
Prediction plot for the theoretical daily dietary dose (DDD_survey_) in relation to percentage of investigated fields on which the species was recorded (FO_field_) and per feeding group for all examples. The lines indicate the model predictions from the respective generalized linear mixed models. The shaded area shows the confidence interval of the prediction. Note the different y-axis scales.

Figure 2 shows the relationship between FO_field_ and DDD_survey_ for each feeding group and differentiated between transect counts and scan samplings. The results of the best statistical models showed a significant positive correlation between FO_field_ and DDD_survey_ for all feeding groups and both methods across different crop types (used as random effect; *p* < .05), indicating that both approaches (based on FO_field_ or on DDD_survey_) would yield similar results.

The dataset available for this analysis included 585 data points (361 from transect counts, 244 from scan samplings) and was therefore much larger than the dataset used to assess the correlation between FO_survey_ and PT. Hence, it was possible to differentiate between the feeding groups. However, this represents the overall trend only. In individual cases, a closer examination of species with an FO_field_ below 20% but a high DDD_survey_ relative to their respective feeding group is necessary to identify species that may have been overlooked using the former FO_field_ approach. Table 2 provides an example where FO_field_ and DDD_survey_ correlate well, and the ranking of species is similar with both approaches. Conversely, Table 3 highlights a case where a species with an FO_field_ below 20%, a threshold used in EFSA (2009), shows a DDD_survey_ that is the second highest within its feeding group (species 2). This species might have been previously overlooked and should be considered using the approach proposed here.

**Table 3.** Case II: Results from a focal species study.

	FO_field_ %	FO_survey_ (90^th^ percentile) %	DDD_survey_	PT (90^th^ percentile consumer)
**Species 1**	36.67	100.00	2.21	0.09 (*N* = 20)
**Species 2**	16.67	86.67	2.01	–
**Species 3**	36.67	100.00	1.96	0.77 (*N* = 20)
**Species 4**	66.67	100.00	1.93	1.00 (*N* = 20)

*Note.* Not all species were covered by the percentage of investigated fields on which the species was recorded (FO_field_) >20% criterion. In this example, species 2 was not considered in the former approach, as its FO_field_ was below 20%. However, its theoretical daily dietary dose (DDD_survey_) is the second highest and thus, this species would be considered in the new approach. The species are ranked according to their frequency of occurrence in surveys (FO_survey_). The full table is given in the online supplementary material.

## Discussion

The revised EFSA guidance (2023) emphasizes the need to consider the vulnerability of species to a larger extent and suggests using the DDD as an indicator for high exposure levels in focal species selection. Here, we demonstrate an approach for how this one aspect of vulnerability can be incorporated into FS selection. We propose using FO_survey_, a parameter derived from standard FS studies, as a proxy for the PT in the theoretical DDD calculation to rank species according to their potential vulnerability. For this approach to be valid, we expect a correlation between the FO_survey_ value in FS field studies and the PT values obtained from subsequently conducted radio-tracking studies. This correlation was tested in our analysis based on a pool of 10 field case examples of industry-owned FS and PT study pairs. The general positive correlation between FO_survey_ and PT suggests that our approach is valid. However, the number of data points available for a correlation assessment of FO_survey_ and PT was low, especially as with transect counts and scan samplings, two different methods for the assessment of the occurrence of bird species were used. Potentially, there are additional factors that might play a role in this relationship, e.g., the crop and the feeding group. However, the number of available data points did not allow for the inclusion of these factors in the analysis. The lack of a significant correlation in the case of scan sampling is likely attributable to the low number of data points. Nevertheless, it can be seen as a primary indication. Furthermore, a significant positive relationship between the two parameters for the transect count method was found across a diverse range of different crops, suggesting that the proposed approach to use FO_survey_ as a proxy for the PT in DDD_survey_ calculations is justified. This indicates that the relationship is representative and not specific to a common crop, for example, cereals.

Although our approach requires a large dataset of different FO_survey_ and PT study data for the initial validation, we emphasize that once validated, it can be applied to smaller datasets from well-designed FS studies. The basic prerequisite for a reliable analysis is a sufficient number of surveys. The FO_survey_ values obtained with only few surveys seem not to be a useful surrogate for PTs in the calculation of a DDD_survey_ due to the resulting limited range of FO_survey_ values. A low number of surveys limits the amount of different values in the calculated FO_survey_ and consequently hampers the differentiation needed to rank the species according to their DDD_survey_. Furthermore, the analyses presented here were unbalanced between transect counts and scan sampling in terms of available data to investigate the relationship between FO_survey_ and PT. Additional data would be helpful to confirm a positive correlation.

For the subsequent selection of a set of FS based on the DDD_survey_ approach presented here, there is no cut-off value to determine which species should be selected for the PT study, similar to the criterion FO_field_ < 20% used in the former GD (EFSA, 2009). Such a cut-off value could be useful, as the DDD_survey_ is likely influenced by factors such as the crop and feeding group. However, we do not recommend defining a threshold as a cut-off value, as this risks excluding relevant species if set too high or of creating an unnecessary long list of species if set too low. Instead, we advocate for a case-by-case consideration of the DDD_survey_ ranking of the observed species.

The selection of bird species for a subsequent PT study from the list of observed species must consider different aspects. This process should account for the uncertainty of the data and potential coverage of a species by another more vulnerable species of the same feeding guild. The uncertainty around FO_survey_ depends on the study design and the ecology of the bird species. With an appropriate number of study fields and surveys, FO_survey_ is a good estimator for the time an individual spends in the crop for species associated with a few or even only one field. Due to a small home range, this individual is likely observed during each survey, and a high FO_survey_ correctly represents a high proportion of the individual’s time in the crop. In contrast, species with a relatively large home range covering several fields may alternate between fields of the same crop for foraging. In these cases, the exposure will be underestimated by its FO_survey_, as the individual uses the crop more often than it is observed in the study field. Furthermore, the determination of FO_survey_ is based on the observation of birds that are in most cases not individually recognizable. Hence, a change in the composition of a flock in the field will not be recognized, potentially leading to an overestimation of exposure by the FO_survey_. Field experts experienced in conducting FS studies are generally aware of these traits and should be able to account for them during the selection process. However, these and other traits must be considered in assessing the DDD_survey_ ranking and, finally, the selection of a FS. To address these uncertainties in FO_survey_, a weighted approach could be applied, assigning confidence levels to the DDD_survey_ estimates based on species-specific traits, such as home range size and flock dynamics. An additional conservative approach could involve to include more potential FS candidates initially, allowing for further validation in subsequent stages (e.g., PT or PD studies) to ensure comprehensive risk coverage.

The effectiveness of the proposed DDD_survey_ approach in identifying focal species depends critically on the careful selection of representative study regions and fields during the planning of new FS studies. For this approach to work as intended, it is essential to ensure that all relevant species and their spatial distribution are adequately represented in the study design. This includes selecting study areas that capture the full range of potential species' habitats within the crop of interest. Failure to do so could result in underrepresentation of vulnerable species and consequently an underestimation of risk. This emphasizes the importance of well-designed, comprehensive FS field studies to support the validity of the DDD_survey_ approach. When appropriately applied, this method provides a more nuanced and inclusive framework for FS selection than the approach based solely on FO_field_, which might miss certain species with high potential exposure.

In general, our results indicated that using FO_survey_ as a proxy for PT in DDD_survey_ calculations is a promising approach that effectively considers species vulnerability in terms of its probability of exposure. Further aspects of vulnerability, such as the species’ life history (e.g., number of breeding attempts per year, number of offspring, number of generations per year, time from immaturity to breeding), i.e., the species’ ecological vulnerability, must be taken into account in future developments of the approach, although the latter might be better addressed in population modeling approaches. Nevertheless, with this limitation to the vulnerability, the proposed method is both practical and effective in identifying appropriate FS for the risk assessment. Although FO_survey_ can be a useful indicator for ranking species to determine which should undergo PT studies to refine the risk assessment, it does not replace a PT study per se.

Our proposed approach uses FO_survey_ as a proxy for PT in DDD_survey_ calculations, providing a practical alternative to direct measurements of time spent in crops. However, we acknowledge that PT measurements from radio-tracking studies, as discussed by Ludwigs et al. (2022), reveal significant inter- and intra-individual variability in crop use. This variability can arise from differences in behavior over time and among individuals, influenced by factors such as species ecology, crop type, and breeding status. Although our use of the 90^th^ percentile aims to provide conservative exposure estimates, we recognize that other metrics, such as the mean or median, could also be applied within the same framework. These metrics might provide less conservative estimates and could potentially account for variability differently. However, we expect that with sufficient data, the overall results and conclusions would remain similar regardless of whether the mean or the 90^th^ percentile is used. Incorporating variance in PT values, as highlighted by Ludwigs et al. (2022), and evaluating the impact of using different metrics represent valuable directions for future refinement of risk assessment methods. Although addressing these aspects is beyond the scope of the current study, the flexibility of the proposed method allows for such adaptations depending on specific regulatory requirements or study objectives.

The correlation of FO_field_ and DDD_survey_ values evaluated whether the proposed DDD_survey_ approach results in a different selection of FS than the former FO_field_ approach. The positive correlation between FO_field_ and the newly derived DDD_survey_ suggests that the new approach would lead to similar FS candidates as the former approach based on EFSA (2009) selection criteria. However, we identified some cases of species with a low FO_field_ but a relatively high DDD_survey_ value (Table 3, species 2). These species, previously neglected by the FO_field_-based approach, might need further consideration as potential FS candidates following the new DDD_survey_ approach. Such bird species may have a restricted distribution but use the crop extensively when present. Apart from these cases, the former FO_field_ approach appears to be a pragmatic criterion.

Because it is crucial that the species selected for the risk assessment of plant protection products is indicative of the broader group and protective for all other species within this respective feeding guild, different approaches have been presented previously as to how identification could be conducted. These include literature reviews, expert judgement based on land-use data, species distribution models and, of course, generic focal species studies (e.g., Orioli et al., 2022; Vallon et al., 2018; Dietzen et al., 2014). However, all these approaches considering the frequency of occurrence in fields in FS studies are based on the EFSA birds and mammals’ guidance of 2009 and the corresponding prevalence criteria. Here, we provide a first proposal for a harmonized approach following the revised EFSA guidance (EFSA et al., 2023).

As the revised EFSA guidance document (EFSA et al., 2023) lacks sufficient details on how focal species should be selected, this study proposes a first initiative towards a harmonized approach for focal species selection. Based on the outcome of this study, we encourage further discussion on focal species selection for higher tier risk assessments.

## Supplementary Material

vjae048_Supplementary_Data

## Data Availability

Summarized data are provided in the article and supporting information. The raw data are the property of the respective companies and not publicly available. For more information, contact corresponding author Steven Kragten (steven.kragten@syngenta.com).

## References

[vjae048-B1] Bibby C. , Burgess N. D., Hill D. A., Mustoe S. (2000). Bird census techniques (2nd ed.). Academic Press.

[vjae048-B2] Bonneris E. , Gao Z., Prosser A., Barfknecht R. (2018). Selecting appropriate focal species for assessing the risk to birds from newly drilled pesticide-treated winter cereal fields in France. Integrated Environmental Assessment and Management, 15, 422–436. 10.1002/ieam.4112 PMC6850368 30515968

[vjae048-B3] Brooks M. E. , Kristensen K., Benthem K. J. V., Magnusson A., Berg C. W., Nielsen A., Skaug H. J., Mächler M., Bolker B. M. (2017). glmmTMB balances speed and flexibility among packages for zero-inflated generalized linear mixed modeling. The R Journal, 9, 378–400. 10.32614/RJ-2017-066

[vjae048-B4] Cramp S. (1988). Handbook of the birds of Europe, the Middle East and North Africa. The birds of the western Palearctic (Vol. 5). Oxford University Press.

[vjae048-B5] Cramp S. , Perrins C. M. (1994). Handbook of the birds of Europe the Middle East and North Africa. The birds of the western Palearctic (Vol. 8). Oxford University Press.

[vjae048-B6] Dietzen C. , Edwards P. J., Wolf C., Ludwigs J. D., Luttik R. (2014). Focal species of birds in European crops for higher tier pesticide risk assessment. Integrated Environmental Assessment and Management, 10, 247–259. 10.1002/ieam.1487 24129982

[vjae048-B7] Dunning J. B. (Ed.). (2008). CRC Handbook of avian body masses (2nd ed.). CRC Press.

[vjae048-B8] European Food Safety Authority (EFSA). (2009). Risk assessment for birds and mammals. EFSA Journal, 7, 1438. Article 1438. 10.2903/j.efsa.2009.1438 40123698 PMC11926626

[vjae048-B9] European Food Safety Authority (EFSA), Aagaard, A., Berny, P., Chaton, P. F., Antia, A. L., McVey, E., Arena, M., Fait, G., Ippolito, A., Linguadoca, A., Sharp, R., Theobald, A., & Brock, T. (2023). Risk assessment for birds and mammals. EFSA Journal, 21, Article e07790. 10.2903/j.efsa.2023.7790

[vjae048-B10] European Food Safety Authority (EFSA) Scientific Committee. (2016). Recovery in environmental risk assessment at EFSA. EFSA Journal, 14, Article 4313. 10.2903/j.efsa.2016.4313 PMC11847968 40007845

[vjae048-B11] Holland J. M. , Hutchison M. A. S., Smith B., Aebischer N. J. (2006). A review of invertebrates and seed-bearing plants as food for farmland birds in Europe. Annals of Applied Biology, 148, 49–71. 10.1111/j.1744-7348.2006.00039.x

[vjae048-B12] Ludwigs J. D. , Ebeling M., Fredricks T. B., Murfitt R. C., Kragten S. (2017). Appropriate exposure estimated for wildlife risk assessment of crop production products based on continuous radio telemetry: a case study with woodpigeons. Environmental Toxicology and Chemistry, 36, 1270–1277. 10.1002/etc3656 27753137

[vjae048-B13] Ludwigs J. D. , Ebeling M., Haaf S., Kragten S. (2022). Assessing the portion of diet taken by birds and mammals from a pesticide-treated area—Proposal for a joint way forward. Environmental Toxicology and Chemistry, 41, 1344–1354. 10.1002/etc5311 35188295 PMC9313892

[vjae048-B14] Orioli V. , Caffi A., Marchetto F., Dondina O., Bani L. (2022). Quantitative selection of focal birds and mammals in higher-tier risk assessment: An approach to rice cultivations. Integrated Environmental Assessment and Management, 18, 1020–1034. 10.1002/ieam.4535 34636488 PMC9298216

[vjae048-B15] R Core Team (2023). *R: A language and environment for statistical computing*. R Foundation for Statistical Computing. https://www.R-project.org/

[vjae048-B16] Vallon M. , Dietzen C., Laucht S., Ludwigs J. D. (2018). Focal species candidates for pesticide risk assessment in European rice fields: A review. Integrated Environmental Assessment and Management, 14, 537–551. 10.1002/ieam.4054 29691977

