# Supplementary material for: A proposal on bird focal species selection for higher tier risk assessments of plant protection products in the EU
Source: Integr Environ Assess Manag. 2025 Jan 6;21(3):649–56. doi: 10.1093/inteam/vjae048 (PMC12047016; doi:10.1093/inteam/vjae048)
Supplement: vjae048_Supplementary_Data [file vjae048_supplementary_data.zip › vjae048_Supplementary_Data/Supplements 1 - studies overview - feeding groups - full tables.docx]

**Supplements 1 to ‘A proposal on bird focal species selection for higher tier risk assessments of plant protection products in the EU’**

by Gießing, Benedikt; Kragten, Steven; Hotopp, Ines; Russ, Anja; Fan, Marie; Sprenger, Dennis; Weyers, Arnd; Wolf, Christian

**Full tables**

**ESM Table 1: Overview of paired focal species and PT studies regarding the EU regulatory zone, crop and growth stage (BBCH) in which they were conducted.**

| Regulatory Zone | Crop | BBCH |
| --- | --- | --- |
| South | spinach | 0 - 9 |
| South | vineyard | 0 - 75 |
| South | citrus | 0 - 75 |
| South | pome fruit | 0 - 79 |
| Central | leafy vegetables | 10 - 49 |
| Central | maize | 0 - 19 |
| Central | oilseed rape | > 96, 0 - 9 |
| Central | pome fruit | 7 - 81 |
| Central | pome fruit | 51 - 79 |
| Central | cereals | 0 - 83 |

ESM Table 2: Food energy and RUD of food items according to EFSA (2023), assigned feeding groups and designated feeding guilds.

| FE Food item | RUD Food item | Feeding group | Feeding guild |
| --- | --- | --- | --- |
| Grasses and cereal shoots | Monocotyledon leaves | herbs and leaves | herbivorous |
| Non-grass herbs | Dicotyledon leaves | herbs and leaves | herbivorous |
| Cereal seeds | Weed seeds/ Crop seeds | grains and seeds | granivorous |
| Weed seeds | Weed seeds/ Crop seeds | grains and seeds | granivorous |
| Arthropods (including caterpillars) | Foliar-dwelling arthropods | flying and foliage dwelling insects | insectivorous |
| Soil invertebrates | Ground-dwelling arthropods | ground dwelling invertebrates | insectivorous |
| Arthropods (including caterpillars) | Flying insects | flying and foliage dwelling insects | insectivorous |

**Case I – full table: All species selected in the newly proposed approach were likely covered by the previously selected focal species.** The species ranked highest by the former FO_field_ > 20% criterion also ranked highest in the DDD_survey_ approach and thus, likely, all relevant species were already selected as focal species.

|  | FO_survey_  (90^th^ percentile) [%] | FO_field_  [%] | DDD_survey_ | PT  (90^th^ percentile consumer) |  |
| --- | --- | --- | --- | --- | --- |
| Species 1 | 97.04 | 100.00 | 8.19 | 0.35 (n=20) |  |
| Species 2 | 81.19 | 100.00 | 7.82 | 0.39 (n=20) |  |
| Species 3 | 61.30 | 75.00 | 6.54 | 0.19 (n=20) |  |
| Species 4 | 49.97 | 25.00 | 6.32 | - |  |
| Species 5 | 28.58 | 37.50 | 2.71 | - |  |
| Species 6 | 2.70 | 12.50 | 0.30 | - |  |
| Species 7 | 1.35 | 12.50 | 0.14 | - |  |
| Species 8 | 1.35 | 12.50 | 0.13 | - |  |

**Case II – full table: Not all species were covered by the FO_field_ > 20% criterion.** Species 2 was not considered with the former approach, as its FO_field_ was below 20%. However, its DDD_survey_ is the second highest and thus, this species would be considered in the new approach.

|  | FO_survey_  (90^th^ percentile) [%] | FO_field_  [%] | DDD_survey_ | PT  (90^th^ percentile consumer) |
| --- | --- | --- | --- | --- |
| Species 1 | 100.00 | 36.67 | 2.21 | 0.09 (n=20) |
| Species 2 | 86.67 | 16.67 | 2.01 | - |
| Species 3 | 100.00 | 36.67 | 1.96 | 0.77 (n=20) |
| Species 4 | 100.00 | 66.67 | 1.93 | 1.00 (n=20) |
| Species 5 | 100.00 | 63.33 | 1.80 | - |
| Species 6 | 66.67 | 26.67 | 1.51 | - |
| Species 7 | 56.67 | 13.33 | 1.50 | - |
| Species 8 | 66.67 | 40.00 | 1.39 | - |
| Species 9 | 60.00 | 10.00 | 1.39 | - |
| Species 10 | 66.67 | 36.67 | 1.30 | - |
| Species 11 | 100.00 | 56.67 | 1.21 | - |
| Species 12 | 50.00 | 3.33 | 1.02 | - |
| Species 13 | 33.33 | 3.33 | 0.94 | - |
| Species 14 | 33.33 | 10.00 | 0.75 | - |
| Species 15 | 33.33 | 3.33 | 0.70 | - |
| Species 16 | 33.33 | 3.33 | 0.68 | - |
| Species 17 | 33.33 | 6.67 | 0.64 | - |
| Species 18 | 33.33 | 6.67 | 0.63 | - |
| Species 19 | 33.33 | 6.67 | 0.62 | - |
| Species 20 | 66.67 | 13.33 | 0.53 | - |
| Species 21 | 33.33 | 6.67 | 0.47 | - |
| Species 22 | 33.33 | 3.33 | 0.44 | - |
| Species 23 | 33.33 | 3.33 | 0.43 | - |
| Species 24 | 55.00 | 60.00 | 0.39 | - |
| Species 25 | 33.33 | 3.33 | 0.34 | - |
| Species 26 | 33.33 | 6.67 | 0.31 | - |
| Species 27 | 33.33 | 6.67 | 0.18 | - |
| Species 28 | 33.33 | 3.33 | 0.16 | - |

**Reference**

European Food Safety Authority (EFSA), Aagaard, A., Berny, P., Chaton, P. F., Antia, A. L., McVey, E., Arena, M., Fait, G., Ippolito, A., Linguadoca, A., Sharp, R., Theobald, A., & Brock, T. (2023). Risk assessment for Birds and Mammals. *EFSA Journal, 21*(2), Article e07790. <https://doi.org/10.2903/j.efsa.2023.7790>
