# Supplementary material for: A proposal on bird focal species selection for higher tier risk assessments of plant protection products in the EU
Source: Integr Environ Assess Manag. 2025 Jan 6;21(3):649–56. doi: 10.1093/inteam/vjae048 (PMC12047016; doi:10.1093/inteam/vjae048)
Supplement: vjae048_Supplementary_Data [file vjae048_supplementary_data.zip › vjae048_Supplementary_Data/Supplements 2 - statistical analysis.docx]

**Supplements 2 to ‘A proposal on bird focal species selection for higher tier risk assessments of plant protection products in the EU’**

by Gießing, Benedikt; Kragten, Steven; Hotopp, Ines; Russ, Anja; Fan, Marie; Sprenger, Dennis; Weyers, Arnd; Wolf, Christian

**Statistical analysis**

Here, we present the statistical methods used in the analysis. The comparison of PT and FO_survey_ was limited due to the rather small data set. The data set for the comparison between DDD_survey_ and FO_field_, is much more suitable for an adequate analysis due to the higher number of data points which allow the inclusion of more fixed and random effects that allow a more refined statistical analysis.

We used generalized linear models (GLMs) or generalized linear mixed models (GLMMs) to handle non-normally distributed data. A GLMM can include random effects, which can help to reduce the background variability. Some factors might influence the relationship in both cases (PT vs. FO_survey_ and DDD_survey_ vs. FO_field_) such as the survey method, the feeding guild, but also the crop, or the BBCH stage. Especially for the second comparison, DDD_survey_ vs. FO_field_, the feeding group has to be included as the different food items have different RUDs, resulting in different magnitudes of value for DDD_survey_ between feeding groups_._ As the inclusion of this factor in an interaction term with FO_field_ led to too high variance inflation factors (VIFs), we considered separate models for each of the four feeding guilds. Further details are provided below.

**PT ~ FO_survey_**

The data set available for the statistical analysis of the relationship between PT and FO_survey_ is small. There are 35 data points obtained using the transect count method, and nine using scan sampling. The scan sampling method seems more promising as the number of surveys used to calculate FO_survey_ is usually higher than with transect counts, but the data set here is especially small.

There are many factors that might play a role in this relationship, e.g. the crop and the feeding guild. However, the size of the data set does not allow to include these factors as fixed effects when using GLMMs. Including them as random effects, however, might reduce the variability and allow for a more precise analysis.

**Generalized Linear (mixed) models**

Here, GL(M)Ms were used to assess the correlation between PT and FO_survey_. A GLMM has the advantage that it is suited to handle non-normally distributed data. PT values are between 0 and 1 and thus, a binomial family should be used. The six tested models included no (GLM) to up to two random effects (GLMM) (Table 1). Crop and feeding guild were included as random effects. Both factors might influence the outcome, but due to the small data set, cannot be included as fixed effects. Four out of the five models considered the two methods separately. One model used the pooled data from both methods. The model including both random effects (model 1) was best by Akaike Information Criterion (AIC). The results of this model and of the model without any random effects but distinguishing between the methods (model 3) are displayed here.

Table 1: Model formulas of the tested GL(M)Ms for the comparison between PT and FO_survey_. All six models use a binomial family. Model 1 includes two random effects and considers the methods separately. Model 2 does not include random effects and pools the data of both methods together. Model 3 does not include any random effects and considers the methods separately. Models 4 and 5 consider the methods separately and include one random effect each. Model 6 includes the method in general but not in combination with FO_survey_. Model 1 fits the data best according to the AIC.

| **Model** | **Formula** | **AIC** | **Efron R2** |
| --- | --- | --- | --- |
| 1 | PT ~ Method / FO_survey_ (90th % ile) [%] + (1\|Feeding group) + (1\|Crop) | 1211 | 0.6 |
| 2 | PT ~ FO_survey_ (90th %ile) | 1866 | 0.2 |
| 3 | PT ~ Method / FO_survey_ (90th % ile) [%] | 1654 | 0.4 |
| 4 | PT ~ Method / FO_survey_ (90th % ile) [%] + (1\|Feeding group) | 1592 | 0.4 |
| 5 | PT ~ Method / FO_survey_ (90th % ile) [%] + (1\|Crop) | 1283 | 0.6 |
| 6 | PT ~ Method + FO_survey_ (90th % ile) [%] + (1\|Feeding group) + (1\|Crop) | 1286 | 0.6 |

Table 2: Simplified model output of model 1 for the comparison of PT and FO_survey_. For the transect count method, a significant increase of the PT with increasing FO_survey_ could be determined.

| **Term** | **Estimate** | **p-value** |
| --- | --- | --- |
| Scan sampling / FO_survey_ | 0.00 | 0.7283 |
| Transect count / FO_survey_ | 0.03 | 0.0000 |


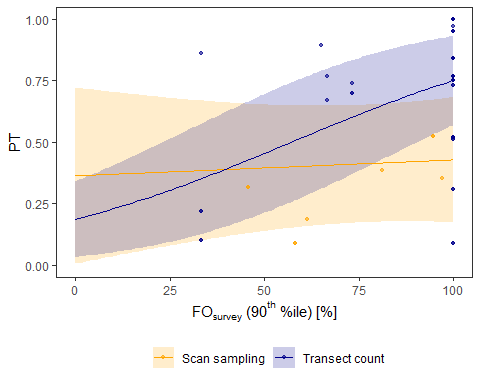


Figure 1: Predicted PT-values for model 1 for the comparison of PT and FO_survey_.

**DDD_survey_ ~ FO_field_**

The data set available for this analysis with 585 data points (361 from transect counts, 244 from scan samplings) is much larger and a more suitable statistical approach that included more fixed effects was possible.

As the different food items in each feeding group result in different RUD-values and thus in a different range of values, models were calculated separately for each feeding group.

**Generalized Linear (mixed) models**

Here, GL(M)Ms were used to assess the correlation between DDD_survey_ and FO_field_. A GLMM has the advantage that it is suited to handle non-normally distributed data. DDD values are between 0 and positive infinity and thus, a gamma family should be used. The four tested models included no (GLM) to up to two random effects (GLMM) (Table 3). Crop and example were included as random effects. Both factors might influence the outcome, but due to the high number of different crops in the data set (8 different crops in 10 cases), cannot be included as fixed effects. Three out of the four models considered the two methods separately. One model used the pooled data from both methods.

Species feeding on grains and seeds

Table 3: Model formulas of the tested GL(M)Ms for the comparison between DDD_survey_ and FO_field_ for species feeding on grains and seeds. All models used a gamma family with log link. Model 1 includes two random effects and considers the methods separately. Modes 2 includes one random effect and considers the methods separately. Model 3 does not include any random effects and considers the methods separately. Model 4 does not consider the methods separately and does not include any random effect. Models 1, 2 and 3 fit the data best according to the AIC.

| **Model** | **Formula** | **AIC** | **Efron R2** |
| --- | --- | --- | --- |
| 1 | DDD_survey_ ~ Method / FO_field_ [%] + (1\|Crop) + (1\|Example) | 575 | 0.5 |
| 2 | DDD_survey_ ~ Method / FO_field_ [%] + (1\|Crop) | 573 | 0.5 |
| 3 | DDD_survey_ ~ Method / FO_field_ [%] | 571 | 0.5 |
| 4 | DDD_survey_ ~ FO_field_ [%] | 637 | 0.3 |

Table 4: Simplified model output of model 2 for the comparison of DDD_survey_ and FO_field_ for species feeding on grains and seeds. Both methods show a significant relationship between DDD_survey_ and FO_field_.

| **Term** | **Estimate** | **p-value** |
| --- | --- | --- |
| Scan sampling / FO_field_ [%] | 0.03 | 0.0000 |
| Transect count / FO_field_ [%] | 0.02 | 0.0000 |


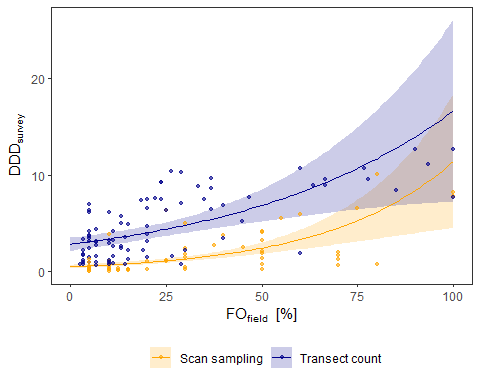


Figure 2: Predicted PT-values for model 2 for the comparison of DDD_survey_ and FO_field_ for species feeding on grains and seeds.

Species feeding on herbs and leaves

Table 5: Model formulas of the tested GL(M)Ms for the comparison between DDD_survey_ and FO_field_ for species feeding on herbs and leaves. All models used a gamma family with log link. Model 1 includes two random effects and considers the methods separately. Model 2 includes one random effects and considers the methods separately. Model 3 does not include any random effects and considers the methods separately. Model 4 does not consider the methods separately and does not include any random effect. Models 1, 2 and 3 fit the data best according to the AIC.

| **Model** | **Formula** | **AIC** | **Efron R2** |
| --- | --- | --- | --- |
| 1 | DDD_survey_ ~ Method / FO_field_ [%] + (1\|Crop) + (1\|Example) | 723 | 0.5 |
| 2 | DDD_survey_ ~ Method / FO_field_ [%] + (1\|Crop) | 721 | 0.5 |
| 3 | DDD_survey_ ~ Method / FO_field_ [%] | 719 | 0.4 |
| 4 | DDD_survey_ ~ FO_field_ [%] | 775 | 0.2 |

Table 6: Simplified model output of model 2 for the comparison of DDD_survey_ and FO_field_ for species feeding on herbs and leaves. Both methods show a significant relationship between DDD_survey_ and FO_field_.

| **Term** | **Estimate** | **p-value** |
| --- | --- | --- |
| Scan sampling / FO_field_ [%] | 0.03 | 0.0000 |
| Transect count / FO_field_ [%] | 0.02 | 0.0001 |


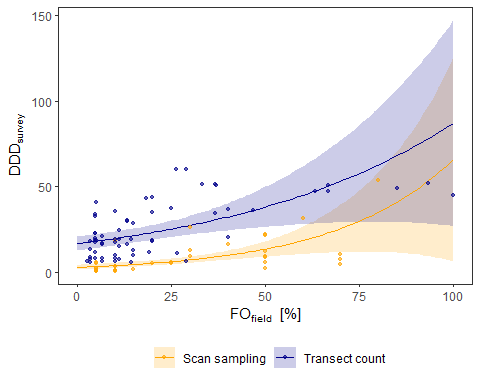


Figure 3: Predicted PT-values for model 2 for the comparison of DDD_survey_ and FO_field_ for species feeding on herbs and leaves.

Species feeding on flying and foliage dwelling insects

Table 7: Model formulas of the tested GL(M)Ms for the comparison between DDD_survey_ and FO_field_ for species feeding on flying and foliage dwelling insects. All models used a gamma family with log link. Model 1 includes two random effects and considers the methods separately. Model 2 includes one random effect and considers the methods separately. Model 3 does not include any random effects and considers the methods separately. Model 4 does not consider the methods separately and does not include any random effect. Models 1, 2 and 3 fit the data best according to the AIC.

| **Model** | **Formula** | **AIC** | **Efron R2** |
| --- | --- | --- | --- |
| 1 | DDD_survey_ ~ Method / FO_field_ [%] + (1\|Crop) + (1\|Example) | 534 | 0.5 |
| 2 | DDD_survey_ ~ Method / FO_field_ [%] + (1\|Crop) | 536 | 0.4 |
| 3 | DDD_survey_ ~ Method / FO_field_ [%] | 553 | 0.3 |
| 4 | DDD_survey_ ~ FO_field_ [%] | 661 | 0.1 |

Table 8: Simplified model output of model 2 for the comparison of DDD_survey_ and FO_field_ for species feeding on flying and foliage dwelling insects. Both methods show a significant relationship between DDD_survey_ and FO_field_.

| **Term** |  | **Estimate** | **p-value** |
| --- | --- | --- | --- |
| Scan sampling / FO_field_ [%] |  | 0.03 | 0.0000 |
| Transect count / FO_field_ [%] |  | 0.01 | 0.0003 |


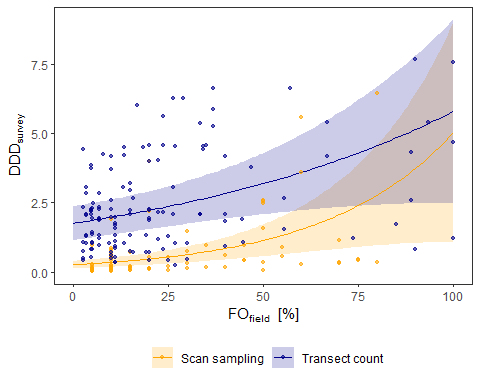


Figure 4: Predicted PT-values for model 2 for the comparison of DDD_survey_ and FO_field_ for species feeding on flying and foliage dwelling insects.

Species feeding on ground dwelling invertebrates

Table 9: Model formulas of the tested GL(M)Ms for the comparison between DDD_survey_ and FO_field_ for species feeding on ground dwelling invertebrates. All models used a gamma family with log link. Model 1 includes two random effects and considers the methods separately. Model 2 includes one random effect and considers the methods separately. Model 3 does not include any random effects and considers the methods separately. Model 4 does not consider the methods separately and does not include any random effect. Models 1, 2 and 3 fit the data best according to the AIC.

| **Model** | **Formula** | **AIC** | **Efron R2** |
| --- | --- | --- | --- |
| 1 | DDD_survey_ ~ Method / FO_field_ [%] + (1\|Crop) + (1\|Example) | 116 | 0.4 |
| 2 | DDD_survey_ ~ Method / FO_field_ [%] + (1\|Crop) | 115 | 0.4 |
| 3 | DDD_survey_ ~ Method / FO_field_ [%] | 116 | 0.3 |
| 4 | DDD_survey_ ~ FO_field_ [%] | 174 | 0.1 |

Table 10: Simplified model output of model 2 for the comparison of DDD_survey_ and FO_field_ for species feeding on ground dwelling invertebrates. Both methods show a significant relationship between DDD_survey_ and FO_field_.

| **Term** | **Estimate** | **p-value** |
| --- | --- | --- |
| Scan sampling / FO_field_ [%] | 0.03 | 0.0000 |
| Transect count / FO_field_ [%] | 0.01 | 0.0002 |


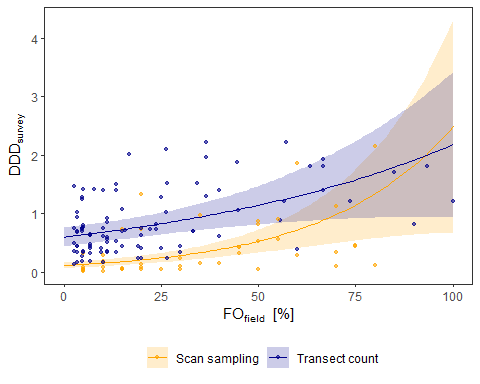


Figure 5: Predicted PT-values for model 2 for the comparison of DDD_survey_ and FO_field_ for species feeding on ground dwelling invertebrates.

**APPENDIX**

Table 11: Full model output of model 1 for the comparison of PT and FO_survey_.

| **summary()** |
| --- |
| Family: binomial ( logit ) Formula:  cbind(success, failure) ~ Method/`FOsurvey (90th %ile) [%]` +   (1 \| `Feeding guild`) + (1 \| Crop) Data: data   AIC BIC logLik deviance df.resid   1211.0 1222.7 -599.5 1199.0 46   Random effects:  Conditional model:  Groups Name Variance Std.Dev.  Feeding guild (Intercept) 0.1542 0.3927   Crop (Intercept) 1.3986 1.1826  Number of obs: 52, groups: Feeding guild, 4; Crop, 7  Conditional model:  Estimate Std. Error z value (Intercept) -0.558328 0.790438 -0.706 MethodTransect count -0.913611 0.656764 -1.391 MethodScan sampling:`FOsurvey (90th %ile) [%]` 0.002707 0.007792 0.347 MethodTransect count:`FOsurvey (90th %ile) [%]` 0.025788 0.001877 13.742  Pr(>\|z\|)  (Intercept) 0.480  MethodTransect count 0.164  MethodScan sampling:`FOsurvey (90th %ile) [%]` 0.728  MethodTransect count:`FOsurvey (90th %ile) [%]` <2e-16 *** --- Signif. codes: 0 '***' 0.001 '**' 0.01 '*' 0.05 '.' 0.1 ' ' 1 |


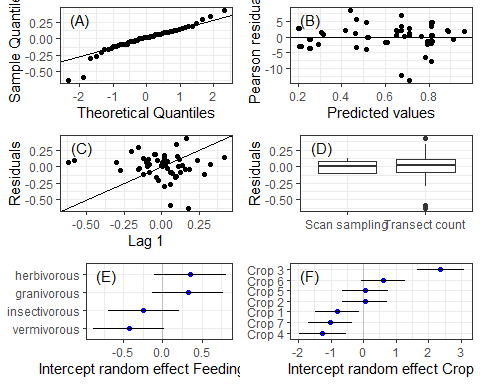


Figure 6: Model diagnostic plots for model 1 chosen for the comparison of PT and FO_survey_. A: Test of normality of residuals. B: Test of Linearity between response and predictors. C: Checking independence. D: Residuals for the method. E: Random effect intercept variance for the crop.

Table 12: VIFs of model 1 chosen for the analysis of the comparison of PT and FO_survey_.

| **Term** | **VIF** | **VIF_CI_low** | **VIF_CI_high** | **SE_factor** | **Tolerance** | **Tolerance_CI_low** | **Tolerance_CI_high** |
| --- | --- | --- | --- | --- | --- | --- | --- |
| Method | 18.92 | 11.97 | 30.29 | 4.35 | 0.05 | 0.03 | 0.08 |
| Method:FOsurvey (90th %ile) [%] | 18.92 | 11.97 | 30.29 | 4.35 | 0.05 | 0.03 | 0.08 |

Table 13: Full model output of model 2 for the comparison of DDD_survey_ and FO_field_ for species feeding on grains and seeds.

| **summary()** |
| --- |
| Family: Gamma ( log ) Formula: DDDsurvey ~ Method/`FOfield [%]` + (1 \| Crop) Data: dt[`Feeding guild` == " grains and seeds"]   AIC BIC logLik deviance df.resid   572.7 590.5 -280.3 560.7 138   Random effects:  Conditional model:  Groups Name Variance Std.Dev.   Crop (Intercept) 5.3e-08 0.0002302 Number of obs: 144, groups: Crop, 7  Dispersion estimate for Gamma family (sigma^2): 0.58   Conditional model:  Estimate Std. Error z value Pr(>\|z\|)  (Intercept) -0.642274 0.171391 -3.747 0.000179 *** MethodTransect count 1.685359 0.210222 8.017 1.08e-15 *** MethodScan sampling:`FOfield [%]` 0.030785 0.004315 7.134 9.77e-13 *** MethodTransect count:`FOfield [%]` 0.017699 0.003673 4.818 1.45e-06 *** --- Signif. codes: 0 '***' 0.001 '**' 0.01 '*' 0.05 '.' 0.1 ' ' 1 |


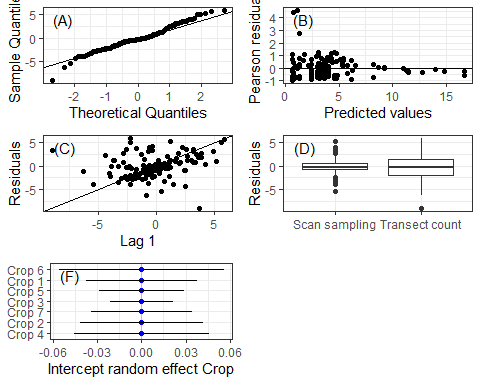


Figure 7: Model diagnostic plots for model 2 chosen for the comparison of DDD_survey_ and FO_field_ for species feeding on grains and seeds. A: Test of normality of residuals. B: Test of Linearity between response and predictors. C: Checking independence. D: Residuals for the method. E: Random effect intercept variance for the crop.

Table 14: VIFs of model 2 chosen for the analysis of the comparison of DDD_survey_ and FO_field_ for species feeding on grains and seeds.

| **Term** | **VIF** | **VIF_CI_low** | **VIF_CI_high** | **SE_factor** | **Tolerance** | **Tolerance_CI_low** | **Tolerance_CI_high** |
| --- | --- | --- | --- | --- | --- | --- | --- |
| Method | 2.64 | 2.09 | 3.45 | 1.62 | 0.38 | 0.29 | 0.48 |
| Method:FOfield [%] | 2.64 | 2.09 | 3.45 | 1.62 | 0.38 | 0.29 | 0.48 |

Table 15: Full model output of model 2 for the comparison of DDD_survey_ and FO_field_ for species feeding on herbs and leaves.

| **summary()** |
| --- |
| Family: Gamma ( log ) Formula: DDDsurvey ~ Method/`FOfield [%]` + (1 \| Crop) Data: dt[`Feeding guild` == " herbs and leaves"]   AIC BIC logLik deviance df.resid   721.0 736.5 -354.5 709.0 93   Random effects:  Conditional model:  Groups Name Variance Std.Dev.  Crop (Intercept) 0.007198 0.08484  Number of obs: 99, groups: Crop, 7  Dispersion estimate for Gamma family (sigma^2): 0.465   Conditional model:  Estimate Std. Error z value Pr(>\|z\|)  (Intercept) 1.000797 0.252543 3.963 7.41e-05 *** MethodTransect count 1.808157 0.284498 6.356 2.08e-10 *** MethodScan sampling:`FOfield [%]` 0.031889 0.006580 4.847 1.26e-06 *** MethodTransect count:`FOfield [%]` 0.016582 0.004297 3.859 0.000114 *** --- Signif. codes: 0 '***' 0.001 '**' 0.01 '*' 0.05 '.' 0.1 ' ' 1 |


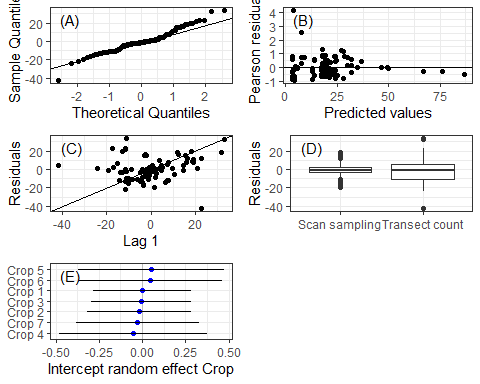


Figure 8: Model diagnostic plots for model 2 chosen for the comparison of DDD_survey_ and FO_field_ for species feeding on herbs and leaves. A: Test of normality of residuals. B: Test of Linearity between response and predictors. C: Checking independence. D: Residuals for the method. E: Residuals for the feeding guild. F: Random effect intercept variance for the crop.

Table 16: VIFs of model 2 chosen for the analysis of the comparison of DDD_survey_ and FO_field_ for species feeding on herbs and leaves.

| **Term** | **VIF** | **VIF_CI_low** | **VIF_CI_high** | **SE_factor** | **Tolerance** | **Tolerance_CI_low** | **Tolerance_CI_high** |
| --- | --- | --- | --- | --- | --- | --- | --- |
| Method | 3.00 | 2.27 | 4.16 | 1.73 | 0.33 | 0.24 | 0.44 |
| Method:FOfield [%] | 3.00 | 2.27 | 4.16 | 1.73 | 0.33 | 0.24 | 0.44 |

Table 17: Full model output of model 2 for the comparison of DDD_survey_ and FO_field_ for species feeding on flying and foliage dwelling insects.

| **summary()** |
| --- |
| Family: Gamma ( log ) Formula: DDDsurvey ~ Method/`FOfield [%]` + (1 \| Crop) Data: dt[`Feeding guild` == "flying and foliage dwelling insects"]   AIC BIC logLik deviance df.resid   535.8 556.0 -261.9 523.8 208   Random effects:  Conditional model:  Groups Name Variance Std.Dev.  Crop (Intercept) 0.09253 0.3042  Number of obs: 214, groups: Crop, 7  Dispersion estimate for Gamma family (sigma^2): 0.666   Conditional model:  Estimate Std. Error z value Pr(>\|z\|)  (Intercept) -1.351834 0.264586 -5.109 3.23e-07 *** MethodTransect count 1.915267 0.318253 6.018 1.77e-09 *** MethodScan sampling:`FOfield [%]` 0.029675 0.004470 6.638 3.17e-11 *** MethodTransect count:`FOfield [%]` 0.011926 0.003266 3.651 0.000261 *** --- Signif. codes: 0 '***' 0.001 '**' 0.01 '*' 0.05 '.' 0.1 ' ' 1 |


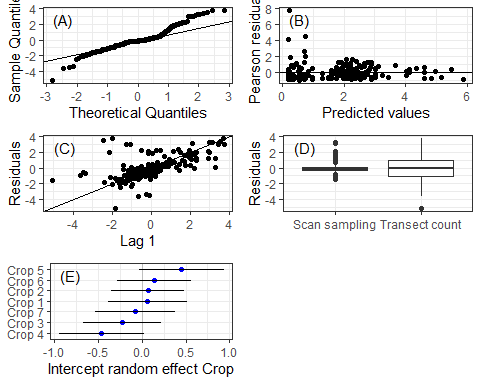


Figure 9: Model diagnostic plots for model 2 chosen for the comparison of DDD_survey_ and FO_field_ for species feeding on flying and foliage dwelling insects. A: Test of normality of residuals. B: Test of Linearity between response and predictors. C: Checking independence. D: Residuals for the method. E: Random effect intercept variance for the crop.

Table 18: VIFs of model 2 chosen for the analysis of the comparison of DDD_survey_ and FO_field_ for species feeding on flying and foliage dwelling insects.

| **Term** | **VIF** | **VIF_CI_low** | **VIF_CI_high** | **SE_factor** | **Tolerance** | **Tolerance_CI_low** | **Tolerance_CI_high** |
| --- | --- | --- | --- | --- | --- | --- | --- |
| Method | 1.26 | 1.12 | 1.56 | 1.12 | 0.79 | 0.64 | 0.89 |
| Method:FOfield [%] | 1.26 | 1.12 | 1.56 | 1.12 | 0.79 | 0.64 | 0.89 |

Table 19: Full model output of model 2 for the comparison of DDD_survey_ and FO_field_ for species feeding on ground dwelling invertebrates.

| **summary()** |
| --- |
| Family: Gamma ( log ) Formula: DDDsurvey ~ Method/`FOfield [%]` + (1 \| Crop) Data: dt[`Feeding guild` == "ground dwelling invertebrates"]   AIC BIC logLik deviance df.resid   115.3 133.2 -51.6 103.3 141   Random effects:  Conditional model:  Groups Name Variance Std.Dev.  Crop (Intercept) 0.03084 0.1756  Number of obs: 147, groups: Crop, 7  Dispersion estimate for Gamma family (sigma^2): 0.551   Conditional model:  Estimate Std. Error z value Pr(>\|z\|)  (Intercept) -2.195366 0.227181 -9.664 < 2e-16 *** MethodTransect count 1.680492 0.268734 6.253 4.02e-10 *** MethodScan sampling:`FOfield [%]` 0.031070 0.004865 6.387 1.70e-10 *** MethodTransect count:`FOfield [%]` 0.012923 0.003473 3.721 0.000198 *** --- Signif. codes: 0 '***' 0.001 '**' 0.01 '*' 0.05 '.' 0.1 ' ' 1 |


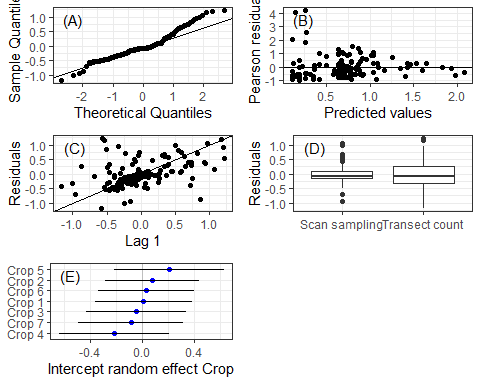


Figure 10: Model diagnostic plots for model 2 chosen for the comparison of DDD_survey_ and FO_field_ for species feeding on ground dwelling invertebrates. A: Test of normality of residuals. B: Test of Linearity between response and predictors. C: Checking independence. D: Residuals for the method. E: Random effect intercept variance for the crop.

Table 20: VIFs of model 2 chosen for the analysis of the comparison of DDD_survey_ and FO_field_ for species feeding on ground dwelling invertebrates.

| **Term** | **VIF** | **VIF_CI_low** | **VIF_CI_high** | **SE_factor** | **Tolerance** | **Tolerance_CI_low** | **Tolerance_CI_high** |
| --- | --- | --- | --- | --- | --- | --- | --- |
| Method | 1.69 | 1.41 | 2.17 | 1.30 | 0.59 | 0.46 | 0.71 |
| Method:FOfield [%] | 1.69 | 1.41 | 2.17 | 1.30 | 0.59 | 0.46 | 0.71 |
